# Evolution of mobility, pain/discomfort, self-care, and mental health in patients with alpha-mannosidosis: an international caregiver and patient survey

**DOI:** 10.1186/s13023-025-03694-4

**Published:** 2025-05-07

**Authors:** Karolina M. Stepien, Sophie Thomas, Julia B. Hennermann, Christina Lampe, Nicole M. Muschol, Maria Juliana Ballesta-Martínez, Jordi Cruz, Mónica López-Rodríguez, Anneliese Barth, Martin Magner, Allan M. Lund, Vasilica Plaiasu, Andrea Ballabeni, Francesca Donà, Heather M. Morgan, Nathalie Guffon

**Affiliations:** 1 Adult Inherited Metabolic Disorders, Salford Care Organisation, Northern Care Alliance NHS Foundation Trust, Salford, UK; 2 The Society for Mucopolysaccharide and Related Diseases, MPS House, Amersham, UK; 3 Villa Metabolica, University Medical Center Mainz, Mainz, Germany; 4 Centre for Rare Diseases, University Hospital of Giessen, Giessen, Germany; 5 University Medical Center Hamburg-Eppendorf, Hamburg, Germany; 6 Hospital Universitario Virgen de la Arrixaca, Murcia, Spain; 7 Asociación MPS-Lisosomales España, Barcelona, Spain; 8 Hospital Universitario Ramón y Cajal, IRYCIS, Madrid, Spain; 9 Instituto Fernandes Figueira /Fiocruz, Rio de Janeiro, Brazil; 10 Department of Pediatrics and Inherited Metabolic Disorders, General University Hospital and First Faculty of Medicine, Charles University, Prague, Czech Republic; 11 Centre for Inherited Metabolic Diseases, Copenhagen University Hospital, Copenhagen, Denmark; 12 Regional Center of Medical Genetics Bucharest, INSMC Alessandrescu-Rusescu, Bucharest, Romania; 13 Chiesi Farmaceutici S.p.A, Parma, Italy; 14 Chiesi USA Inc., Boston, USA; 15 Reference Center for Inherited Metabolic Disorders, Femme Mère Enfant Hospital, Hospices Civils de Lyon, Lyon, France

**Keywords:** Alpha-mannosidosis, Survey, Walking, Pain, Self-care, Mental health

## Abstract

**Background:**

Alpha-mannosidosis is a rare recessive lysosomal storage disorder with progressive multi-systemic impacts. In the absence of standardized monitoring protocols, there is insufficient understanding of disease progression over time. This study explored the evolution of the burden of illness and quality of life (QoL) experienced by patients with alpha-mannosidosis via an international patient and caregiver-based survey. The online survey was distributed to adult patients/caregivers of patients ≥ 10 years old. It included visual analogue scales (VAS; timepoints 5 years ago and now), multiple choice, and open text questions. We report a subset of functional and QoL data: walking ability, pain/discomfort, ability to self-care, and mental health.

**Results:**

Analyses include 51 responses from 18 countries: 26 patients were on velmanase alfa enzyme replacement therapy (ERT), seven had been treated with hematopoietic stem cell transplantation (HSCT) and 18 were untreated patients (UP). Over 5 years, VAS scores showed the least decline in walking ability for HSCT patients (+ 0.1 ± 1.9) compared to patients receiving ERT (+ 0.7 ± 1.2) and UP (+ 1.8 ± 2.0). A trend towards improvement in pain was only observed for those on ERT (-0.2 ± 2.0), both for pediatric and adult patients. Ability to self-care improved for patients treated with HSCT (-1.0 ± 1.8) and slightly improved with ERT (-0.3 ± 1.5) but worsened for UP (+ 0.6 ± 0.9). Similarly, a trend towards improvement in mental health scores was observed for patients on ERT (-0.4 ± 2.2).

**Conclusions:**

Alpha-mannosidosis is associated with a substantial and progressive burden in UP, including deterioration in walking ability, pain, self-care and mental health. The survey results suggest that treatment with ERT or HSCT may slow this natural progression of alpha-mannosidosis, with these patients following a different disease trajectory to those solely receiving supportive care. This study could inform the natural pathway of alpha-mannosidosis to recognize patients’ needs, courses of care, and the design of interventional studies.

**Supplementary Information:**

The online version contains supplementary material available at 10.1186/s13023-025-03694-4.

## Background

Alpha-mannosidosis (OMIM 248500) is a rare progressive autosomal recessive lysosomal storage disorder (LSD) caused by pathogenic variants in the *MAN2B1* gene (609458), leading to deficient activity of the alpha-mannosidase enzyme and accumulation of undigested mannose-rich oligosaccharides in cells [1, 2]. Alpha-mannosidosis is present worldwide and its prevalence is estimated at 1:500000 live births [3, 4].

Disease progression and severity are heterogeneous, but most children present with clinical manifestations in their first decade, which progressively worsen over time [5, 6]. Symptoms include facial and skeletal abnormalities, hearing loss, speech difficulties, immunodeficiency with recurrent infections, muscular weakness, ataxia, cognitive impairment, psychiatric manifestations and a potential increased risk of cancer [2, 6–8]. Attenuated forms are associated with slow progression and survival into adulthood; severe forms with rapid progression and death in childhood [5].

There are limited disease-modifying treatments for alpha-mannosidosis, including hematopoietic stem cell transplantation (HSCT) and enzyme replacement therapy (ERT), but most patients only receive supportive care [9, 10]. HSCT may be effective in improving physical manifestations and in preserving neurocognitive function, with promising results in some patients [11–13]. HSCT should be conducted as early as possible to prevent irreversible pathological changes [13]. Although HSCT has yielded positive effects, especially on the central nervous system [13], there are associated morbidity and mortality risks [14]. The first human recombinant form of alpha-mannosidase available for ERT, velmanase alfa, was approved in 2018 in Europe [15] and in 2023 in the United States [16], for the treatment of non-neurologic (Europe) and non-central nervous system (US) manifestations of alpha-mannosidosis. Velmanase alfa significantly reduced serum oligosaccharide concentration in placebo-controlled studies and trends towards improvement were observed in immune, motor and lung functions, and quality of life (QoL) in placebo-controlled and open-label studies [12, 17–21].

Understanding the natural history of alpha-mannosidosis is important, e.g., which clinical symptoms develop over time and what effect these changes have on the patient’s QoL, in order to optimize symptom management and gauge the effectiveness of therapies. Clinical manifestations in patients with alpha-mannosidosis have previously been described in detail but studies have been primarily based on individual case reports, small numbers of patients or short follow-up times [2, 3, 6, 22, 23]. Ongoing efforts such as the SPARKLE registry (EUPAS29038) aim to change that by collecting international long-term prospective standardized data on a large cohort of alpha-mannosidosis patients [5].

A Delphi consensus to integrate recommendations for coordination of care for alpha-mannosidosis has been recently undertaken to help address the current lack of standardized practice and monitoring protocols [24]. The rarity of the disease and the difficulty in accessing patients, challenge the collection of robust datasets, especially natural history data from those who have not received targeted therapies. Caregiver proxy can measure patient-reported outcomes in young or cognitively impaired patients not able to report changes in symptoms or QoL domains themselves. The aim of this study was to bridge the gap between clinical assessments and the natural history of alpha-mannosidosis from the caregivers and patients’ perspective by exploring the evolution of the burden of illness and QoL experienced by patients with alpha-mannosidosis through a patient and caregiver-based survey. Here we present the first set of results from the study, focusing on changes perceived by respondents in patients’ walking ability, pain and discomfort, ability to self-care, and mental health at the time of the survey, and 5 years prior.

## Methods

### Participants

LSD/mucopolysaccharidoses (MPS) patient organizations (POs) and clinicians from specialized centers were identified through the UK MPS Society and Rare Disease Research Partners (RDRP) (Supplementary file 1). Thirty-one clinicians and six POs participated in the study and were emailed a link/QR code to access the online survey to share with patients and caregivers who met the eligibility criteria (Table 1). Survey responses were anonymous to clinicians, POs and the sponsor.

**Table 1:** Eligibility criteria

Eligibility criteria
Respondents had to be ≥ 18 years old to complete the survey and provide informed consent. The survey was open to: • Patients who were ≥ 18 years old with alpha-mannosidosis • Caregivers* responding on behalf of patients ≥ 10 years old • Patients who had received a HSCT, those treated with ERT at the time of the study and patients who had received no specific treatment (no HSCT or ERT)

^*^ *A caregiver was defined as the person who was responsible for the patient daily or at least three days per week. Caregivers with more than one child with alpha-mannosidosis were asked to complete a separate survey for each child.*

*ERT = enzyme replacement therapy; HSCT = hematopoietic stem cell transplant*

### Survey design

The online survey was available November 2022 to February 2023 in 13 languages: Czech, Danish, English, Finnish, French, German, Italian, Lithuanian, Portuguese (Brazil), Romanian, Russian, Spanish, and Turkish. A participation information sheet and informed consent were provided at the start of the survey. Screening questions confirmed the eligibility criteria. Questions related to changes in the burden of illness and QoL of the patient (respondent: patient or caregiver proxy) and about the QoL of the caregiver (respondent: caregivers) over time (10 years ago, 5 years ago and now) and included visual analogue scales (VAS), multiple choice, and open text questions to provide both quantitative and qualitative data. The survey was hosted on the on-line Qualtrics^XM^ platform.

### Analysis

The survey collected data on eleven functional and QoL patient domains and five QoL caregiver domains. Here we report data on the subset of patient domains which are most frequently collected in clinical trials - walking ability, pain or discomfort, self-care, and mental health to provide insights into data gaps from clinical trial observations. In this study, mental health refers to issues such as depression, anxiety, or aggression, and not to cognitive ability/function. Caregiver data will be the subject of a subsequent publication. Categorical variables were reported as frequencies and continuous variables as descriptive statistics, including VAS scores. A VAS is an instrument used to measure a characteristic across a continuum of values. Respondents can score the severity of the variable by selecting a number from 0 – 10 on a horizontal line with extreme descriptors at each end, for example, 0 “no pain” and 10 “worst pain possible”. VAS are most useful when investigating change overtime within individuals and an arithmetic mean can be calculated [25, 26]. Data from 10 years ago were excluded from analysis to minimize reliability issues when recalling patients’ status. Mean change in VAS scores over time was calculated as the difference in VAS scores between 5 years ago and at the time of the survey (referred to as ‘now’), where a positive mean change in scores (higher VAS score) signified a trend towards worsening, and a negative mean change in scores (lower VAS score) signified a trend towards improvement. Mean changes in VAS scores were considered indicative of descriptive trends rather than significant evidence of differences. Changes in VAS scores < 1.0 are described as ‘slight’ improvement/worsening, otherwise they are referred to as trends towards improvement/worsening. Data were assessed for patients on ERT, patients who had received HSCT and untreated patients (UP). Results for patients receiving ERT were further assessed by age, pediatric < 16 years old vs adults ≥ 16 years old (16 years of age is the cut-off point used in clinic in some countries to transition patients from pediatric to adult centers), and by the length of time on ERT (< 5 years vs ≥ 5 years). These subgroup analyses were not performed for patients treated with HSCT or UP due to the small sample size of the overall HSCT group of patients and of the pediatric UP. Quantitative analysis was performed using STATA (StataCorp. 2023. Stata Statistical Software: Release 18). Qualitative analysis of free text responses used an inductive thematic approach [27].

### Ethical considerations

RDRP conducted this survey in accordance with the British Healthcare Business Intelligence Association’s (BHBIA) Legal & Ethical Guidelines for Market Research [28] and the European Pharmaceutical Market Research Association (EPHMRA). In line with these standards, this market research did not require review of a Research Ethics Committee in the UK because it falls outside the remit of the Research Governance Framework. The confidentiality of information gathered during the study is protected in accordance with applicable data protection legislation and BHBIA’s Legal & Ethical Guidelines.

## Results

### Respondents

**Fig. 1:**
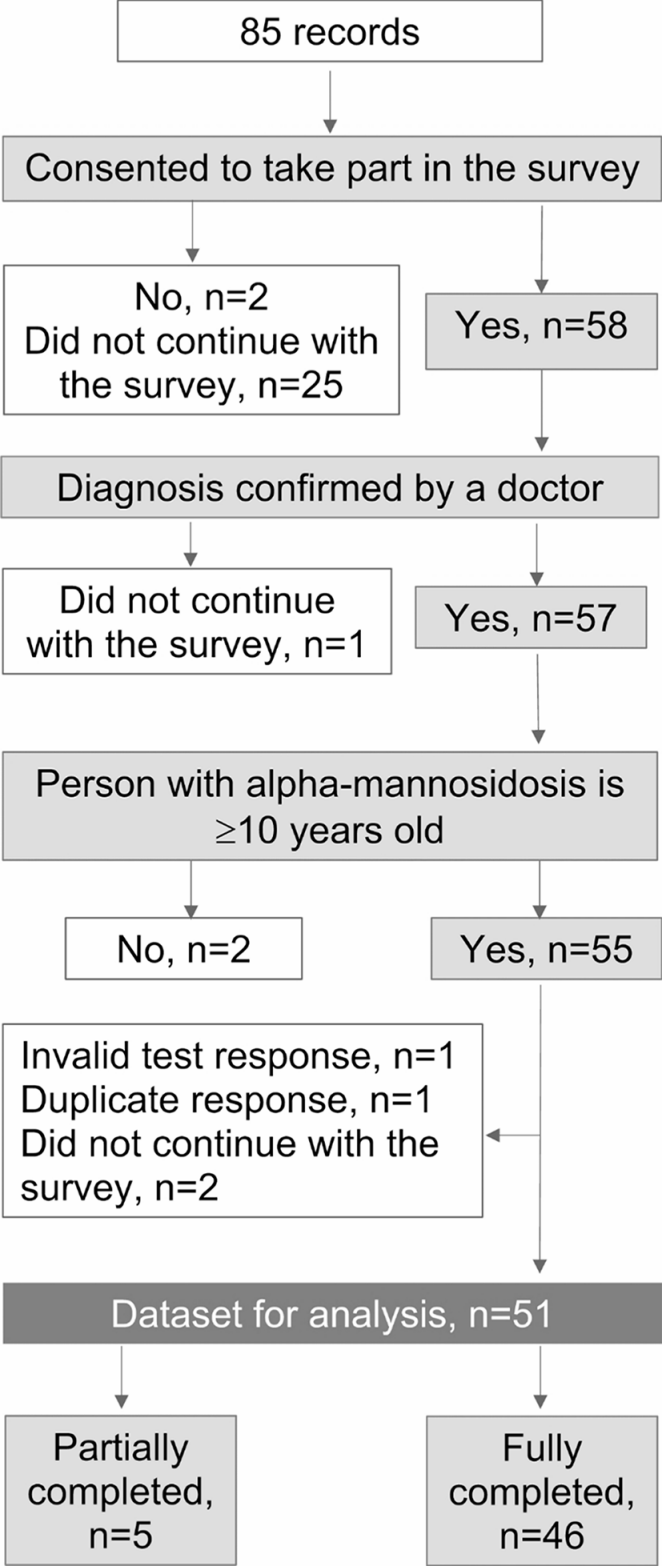
Flow diagram of respondents included in the analysis

Fifty-one respondents provided valid responses: 46 fully completed and five partially completed (Fig. 1). Three respondents were a person with alpha-mannosidosis (all females, one had received HSCT and two were on ERT) and 48 the caregiver of a person with alpha-mannosidosis (mothers = 44; fathers = 4). Responses were received from patients living in 18 countries: Australia, Brazil, Czech Republic, Denmark, Finland, France, Germany, Ireland, Italy, Lithuania, Netherlands, Romania, Russia, Saudi Arabia, Spain, Turkey, United Kingdom, and USA.

### Patient demographics

From 51 patients (49% female), 26 were on ERT at the time of the survey; seven had received an HSCT and 18 were UP (including two patients who discontinued ERT treatment after six months). All HSCT patients had received only one transplant and none of the patients had received both a HSCT and ERT. Ten patients (7 on ERT and 3 UP) had received the diagnosis because of a confirmed diagnosis of a sibling (6 males, 4 females). Patient demographics are summarized in Table 2.

#### Patients receiving ERT

At the time of the survey, median ages of pediatric patients and adults on ERT were 12.7 years (*n* = 6) and 29.8 years (*n* = 20), respectively. The median ages at symptom presentation and diagnosis were, respectively, 2.4 and 7.7 years for pediatric patients, and 1.3 and 6.1 years for adults. Patients had been receiving ERT for a median of 5.3 years (between 0.3 and 12.5 years).

#### Patients who had received HSCT

Pediatric HSCT patients were a median of 12.2 years old (*n* = 4) and adults 20.7 years old (*n* = 3). The median ages of symptom presentation and diagnosis were, respectively, 1.8 and 4.4 years for pediatric patients, and 0.5 and 1.9 years for adults. Overall, patients received HSCT at a median age of 3.2 years old (median of 10.4 years since HSCT).

#### Untreated patients

Median age of UP (10/18 female) at the time of the survey was 29.2 years, with two pediatric patients. First symptoms and diagnosis were reported to be at an older median age compared to those treated with HSCT and ERT, with the delay between first reported symptoms and diagnosis being the largest of the three groups (UP: 7.9 years; HSCT: 2.1 years; ERT: 5.1 years).

**Table 2:** Patient demographics

		ERT				HSCT			UP		
	All	Pediatrics < 16 years old	Adults ≥ 16 years old	< 5 years on ERT	≥ 5 years on ERT	All	Pediatrics < 16 years old	Adults ≥ 16 years old	All	Pediatrics < 16 years old	Adults ≥ 16 years old
N	26	6	20	13	13	7	4	3	18	2	16
**Sex**											
Males	15	5	10	9	6	3	1	2	8	0	8
Females	11	1	10	4	7	4	3	1	10	2	8
**Age at the time of survey**											
Median	21.1	12.7	29.8	18.6	29.3	13.6	12.2	20.7	29.2	11.8	30.0
Mean ± SD	24.9 ± 10.8	12.8 ± 2.4	28.5 ± 9.6	22.0 ± 10.8	27.8 ± 10.3	15.9 ± 4.9	12.4 ± 0.9	20.7 ± 3.4	26.4 ± 7.8	11.8 ± 0.1	28.3 ± 6.1
Range	10.3–45.7	10.3–15.7	16.3–45.7	10.3–42.2	13.6–45.7	11.4–24.2	11.4–13.6	17.3–24.2	11.7–38.4	11.7–11.8	17.3–38.4
**Age symptoms were first noticed**											
Median	1.5	2.4	1.3	2.0	1.1	0.7	1.8	0.5	2.4	6.5	2.3
Mean ± SD	2.6 ± 4.5	2.6 ± 2.2	2.7 ± 5.0	2.2 ± 1.5	3.0 ± 6.3	2.4 ± 4.0	3.7 ± 5.2	0.6 ± 0.3	5.8 ± 7.2	6.5 ± 5.6	5.7 ± 7.6
Range	0.0–23.5	0.0–5.1	0.0–23.5	0.5–5.1	0.0–23.5	0.0–11.3	0.0–11.3	0.3–1.0	0.3–24.1	2.6–10.5	0.3–24.1
**Age at diagnosis**											
Median	6.6	7.7	6.1	7.0	6.2	2.8	4.4	1.9	10.3	8.3	10.3
Mean ± SD	8.3 ± 7.2	7.3 ± 2.1	8.6 ± 8.1	8.5 ± 7.3	8.0 ± 7.3	3.8 ± 3.0	5.0 ± 3.6	2.1 ± 0.6	11.3 ± 7.0	8.3 ± 3.1	11.7 ± 7.3
Range	0.0–27.2	4.5–9.6	0.0–27.2	1.3–27.2	0.0–25.2	1.3–9.8	1.3–9.8	1.6–2.8	2.6–24.1	6.2–10.5	2.6–24.1
**Age treatment started**											
Median	17.0	8.4	21.5	16.1	21.0	3.2	5.1	2.8			
Mean ± SD	18.9 ± 9.7	9.2 ± 3.3	21.8 ± 9.1	19.8 ± 10.0	17.9 ± 9.7	4.0 ± 2.4	4.9 ± 3.0	2.8 ± 0.8			
Range	5.3–37.5	5.3–14.8	5.7–37.5	7.7–37.5	5.3–34.8	1.6–7.8	1.6–7.8	2.0–3.6			
**Treatment length**											
Median	5.3	2.0	5.9	1.9	10.2	10.4	7.6	17.8			
Mean ± SD	6.0 ± 4.4	3.6 ± 3.8	6.7 ± 4.4	2.2 ± 1.4	9.9 ± 2.5	12.0 ± 6.5	7.5 ± 3.1	17.9 ± 4.2			
Range	0.3–12.5	0.5–10.3	0.3–12.5	0.3–4.7	5.8–12.5	4.3–22.2	4.3–10.4	13.8–22.2			

*Treatment length varies for all patients irrespective of treatment group. Hence*,* data from 5 years ago to now correspond to different follow-up periods in relation to start of treatment of the patients these caregivers care for.*

*ERT = enzyme replacement therapy; HSCT = hematopoietic stem cell transplantation; SD = standard deviation; UP = untreated patients.*

### Walking ability

#### Overall changes

**Fig. 2:**
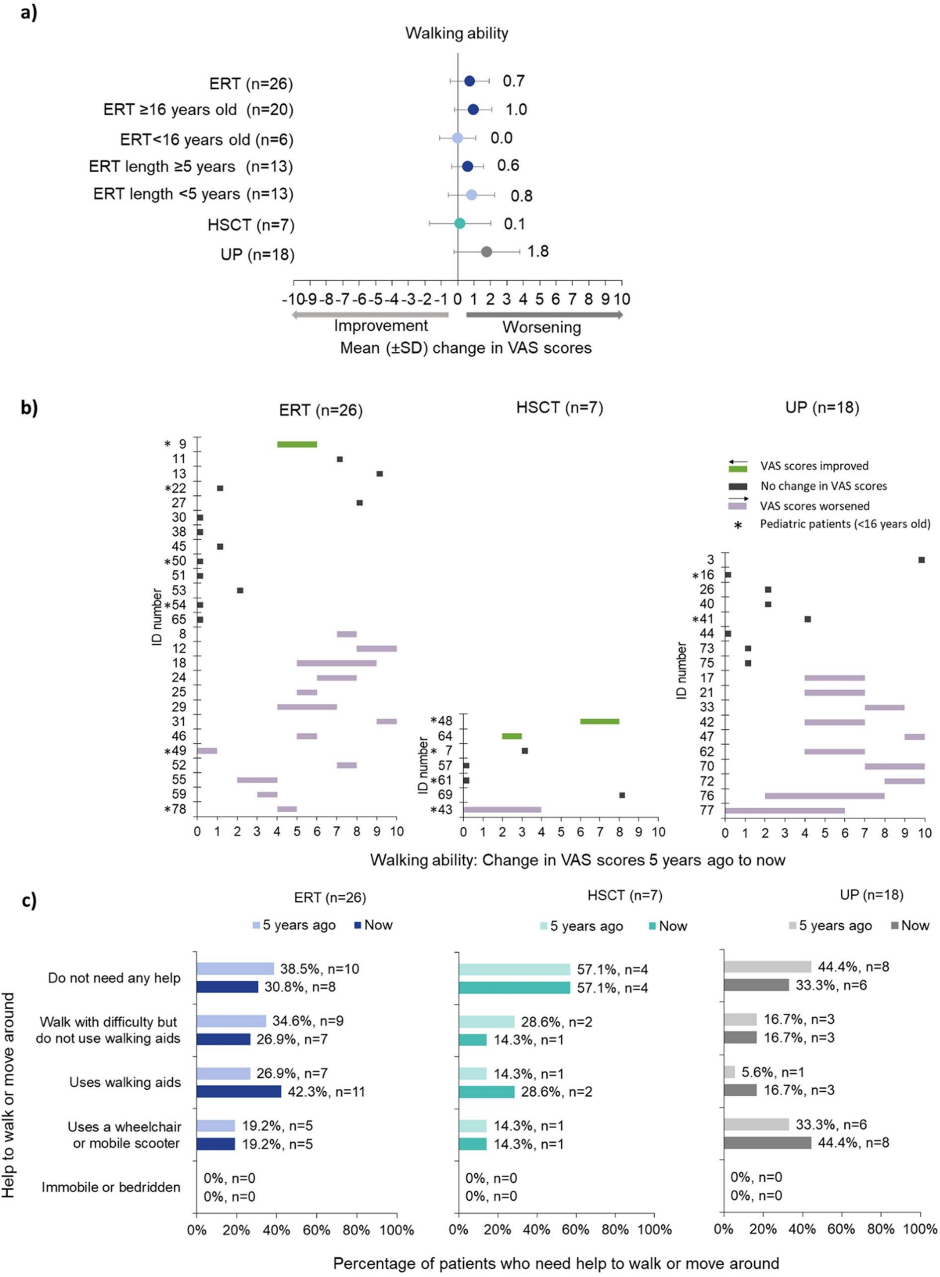
Walking ability. Respondents were asked to rate the person with alpha-mannosidosis’ ability to walk 5 years ago and now on a VAS, where 0 was ‘no problem walking about’ and 10 was ‘unable to walk about’. **(a)** Mean (± SD) change in VAS scores 5 years ago to now, stratified by treatment (ERT, HSCT, UP), pediatric (< 16 years old) vs adults (≥ 16 years old), and length of time receiving ERT (< 5 years; ≥5 years); **(b)** Individual changes in VAS scores for ERT, HSCT, and UP. Green shows improvement, grey no change, and purple worsening/decline; **(c)** Help required to walk or move around 5 years ago and now for patients receiving ERT (*n* = 26), those who had received a HSCT (*n* = 7), and UP (*n* = 18). Respondents could select as many responses as it applied, hence percentages were calculated as number of patients who chose this option over the number of total patients who responded to this question. ERT = enzyme replacement therapy; HSCT = hematopoietic stem cell transplantation; SD = standard deviation; UP = untreated patients; VAS = visual analogue scale

Mean (± SD) VAS walking ability scores 5 years ago were similar for UP (3.8 ± 3.2) and patients treated with ERT (3.8 ± 3.2) and HSCT (3.1 ± 3.6) (Supplementary Table 1). However, decline of walking ability was reported as the largest in UP (+ 1.8 ± 2.0), with HSCT patients declining the least (+ 0.1 ± 1.9) (Fig. 2a).

Within the ERT patient subgroups by age and by duration of ERT treatment, the mean walking ability scores remained the same for pediatric patients on ERT (0.0 ± 1.1) and declined for adults on ERT (+ 1.0 ± 1.1), but less than for the UP (Fig. 2a). Patients receiving ERT for ≥ 5 years experienced a decline in walking ability as did those on ERT for < 5 years (+ 0.6 ± 1.0 and + 0.8 ± 1.4, respectively) (Fig. 2a). Walking ability declined less for those who started ERT at pediatric age (+ 0.2 ± 1.0) than those who started as adults (+ 1.1 ± 1.2) (Supplementary Table 1).

#### Individual changes

According to survey responses, patients reported differing walking ability over time. UP reported having stable walking ability for 44.4% (8/18) of patients and worsened walking ability for the remaining 55.6% (10/18) (Fig. 2b UP).

*“Daughter was able to walk freely until the age of 25*,* at which time she began to be unsteady in her gait. She began using a rolling walker*,* with assistance*,* to walk lengthy distances. At the age of 30 she lost her balance […] and fractured her right humerus. She required surgery to repair her shoulder after which she was no longer able to walk as before. At that time*,* we switched her to a wheelchair because ataxia became an insurmountable obstacle.”* Adult UP.

Walking ability improved for two HSCT patients (change in VAS scores of -1 and -2), remained the same for four, and declined for one (+ 4) (Fig. 2b HSCT).

For those receiving ERT, walking ability improved for one patient, remained the same for 46.2% (12/26) and worsened for 50% (13/26) (Fig. 2b ERT). Out of the 11 patients whose walking ability declined by + 1 or + 2 points on the VAS scale, four were on ERT for ≥ 10 years, and seven for ≤ 6 years (Supplementary Fig. 1a). The two adult patients whose VAS scores declined the most (+ 3, + 4) were on ERT for nearly 6 and 5 years (Supplementary Fig. 1a) and started ERT in their third and fourth decades of life, respectively (Supplementary Fig. 1b). Further examples of the experiences of individual patients, reported by patients or caregivers, are highlighted in Supplementary Table 2).

#### Assistance required to walk or move around

Respondents were asked if patients needed any help walking or moving around 5 years ago and now, and what type of help was needed (i.e., walks with difficulty but does not use walking aids; uses walking aids; uses a wheelchair or mobile scooter; immobile or bedridden; does not need any help). Over time, the number of patients on ERT and UP who were able to walk or move around without help declined. More patients treated with ERT and HSCT needed walking aids such as crutches, canes, walking frames or help from the caregiver at the time of the survey compared to 5 years ago; this was also the case for UP although these patients also had an increased need for wheelchairs or mobile scooters (Fig. 2c).

### Pain or discomfort

Patients and caregivers explained that pain had a large psychological and behavioral impact on patients affecting their mood and restricting socialization and playing with peers. Caregivers noted that due to the limited communication abilities of some patients and the young age of some children, it was not possible to ascertain the level of pain, which was distressing for parents. Some respondents said that although patients might be physically able to walk, pain hindered them from doing so.

#### Overall changes

**Fig. 3:**
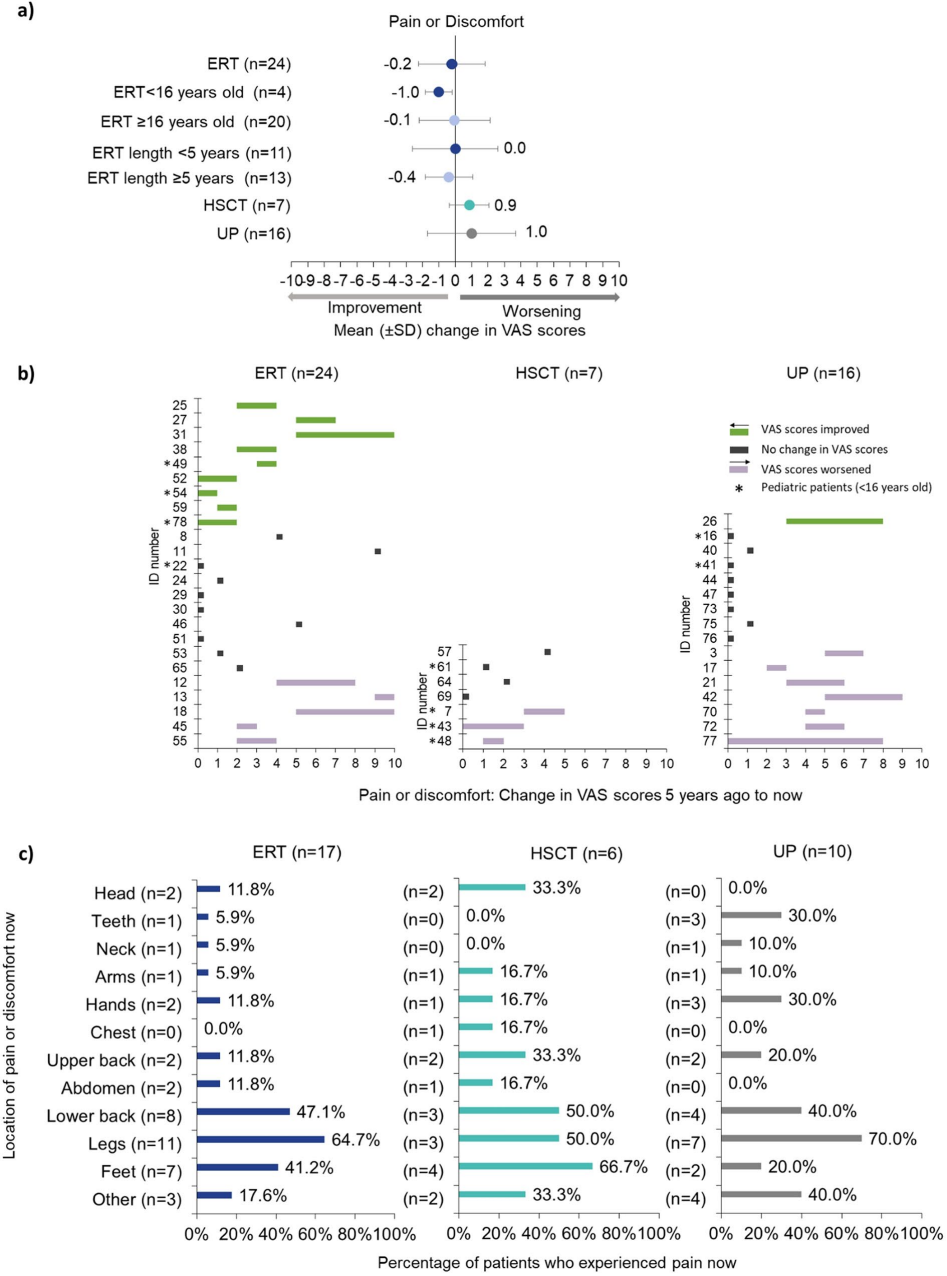
Pain or discomfort. Respondents were asked to rate the person with alpha-mannosidosis’ pain or discomfort now and 5 years ago on a VAS, where 0 was ‘no pain or discomfort’ and 10 was ‘the worst possible pain or discomfort’. **(a)** Mean (± SD) change in VAS scores 5 years ago to now for pain or discomfort, stratified by treatment (ERT, HSCT, UP), pediatric (< 16 years old) vs adults (≥ 16 years old) and length of time receiving ERT (< 5 years; ≥5 years); **(b)** Individual changes in VAS scores for ERT, HSCT, and UP. Green shows improvement, grey no change, and purple worsening/decline; **(c)** Percentage of patients reporting pain or discomfort now in different parts of the body by treatment: ERT (*n* = 17), HSCT (*n* = 6), and UP (*n* = 10). Respondents could select as many responses as applied. Hence, percentages are calculated as number of patients who chose this option over the number of total patients who responded to this question. ERT = enzyme replacement therapy; HSCT = hematopoietic stem cell transplantation; SD = standard deviation; UP = untreated patients; VAS = visual analogue scale

HSCT patients reported the lowest mean VAS score 5 years ago (1.6 ± 1.5; Supplementary Table 1) compared to patients on ERT and UP, a trend that continued for now, however, only patients receiving ERT reported a slight trend towards improvement in pain (-0.2 ± 2.0) (Fig. 3a).

The improvement in the pain/discomfort VAS score was larger in pediatric patients on ERT (-1.0 ± 0.8) than in adults receiving ERT (-0.1 ± 2.2), while the scores remained stable for those on ERT for < 5 years and slightly improved for patients on treatment for ≥ 5 years (-0.4 ± 1.4) (Fig. 3a). There were trends towards improvements in pain over time whether patients had started ERT as adults or at pediatric age (Supplementary Table 1).

#### Individual changes

Overall, from 16 UP, pain improved in one adult (-5), remained stable for 50% (8/16) of patients and worsened for 43.8% (7/16), including one patient who did not experience pain 5 years ago but now reported a VAS score of 8 after falling and dislocating their foot (Fig. 3b UP).

*“Pain in hips is so bad she uses wheelchair. The pain started about 5 years ago. Gets angry due to pain and discomfort. Her life is on hold […] Cannot make plans e.g.*,* holidays or days out.”* Adult UP.

Pain did not improve for any HSCT patient, remained stable for 57.1% (4/7) and worsened for 42.9% (3/7) with a patient reporting no pain 5 years ago but a VAS score of + 3 now because of leg pain (Fig. 3b HSCT).

Pain improved for 37.5% (9/24) of patients on ERT, remained the same for 41.7% (10/24), and worsened for the remaining 20.8% (5/24), with pain reaching a score of 10 now for two patients who described their walking as difficult (Fig. 3b ERT). From the ten patients on ERT whose pain had not changed, two had been on ERT 5–6 years and eight ≥ 10 years (Supplementary Fig. 2a). The two adult patients reporting the highest pain VAS scores (+ 4, + 5) were on ERT for less than half a year and 5 years respectively (Supplementary Fig. 2a) and started ERT in their fourth decade of life (Supplementary Fig. 2b). The adult patient whose pain level improved the most (-5) was on ERT for almost 11 years and started ERT in their fourth decade of life. Examples of individual patient experiences, reported by patients and or caregivers experience, are highlighted in Supplementary Table 2.

#### Location of pain in the body now

Seven patients on ERT, one patient treated with HSCT and six UP reported no pain at the time of the survey, including half of the ten pediatric patients. More than 40% of patients with pain at the time of the survey suffered from pain in the lower back, legs, and feet (Fig. 3c). Pain in the upper back and head was reported by > 30% of HSCT patients, and pain in teeth and hands was noted for 30% of UP patients (Fig. 3c). Those who selected ‘other’ specified they experienced pain in the hips, knees, ankles, and pain after injuries (broken legs/arms and dislocated foot). One ERT pediatric patient located pain in their lower back and legs, and four HSCT pediatric patients in their feet (4/4), legs (2/4), and head (2/4). Some patients reported receiving medication for their pain or discomfort: 7/17 ERT, 2/6 HSCT and 5/10 UP.

### Ability to self-care

#### Overall changes

**Fig. 4:**
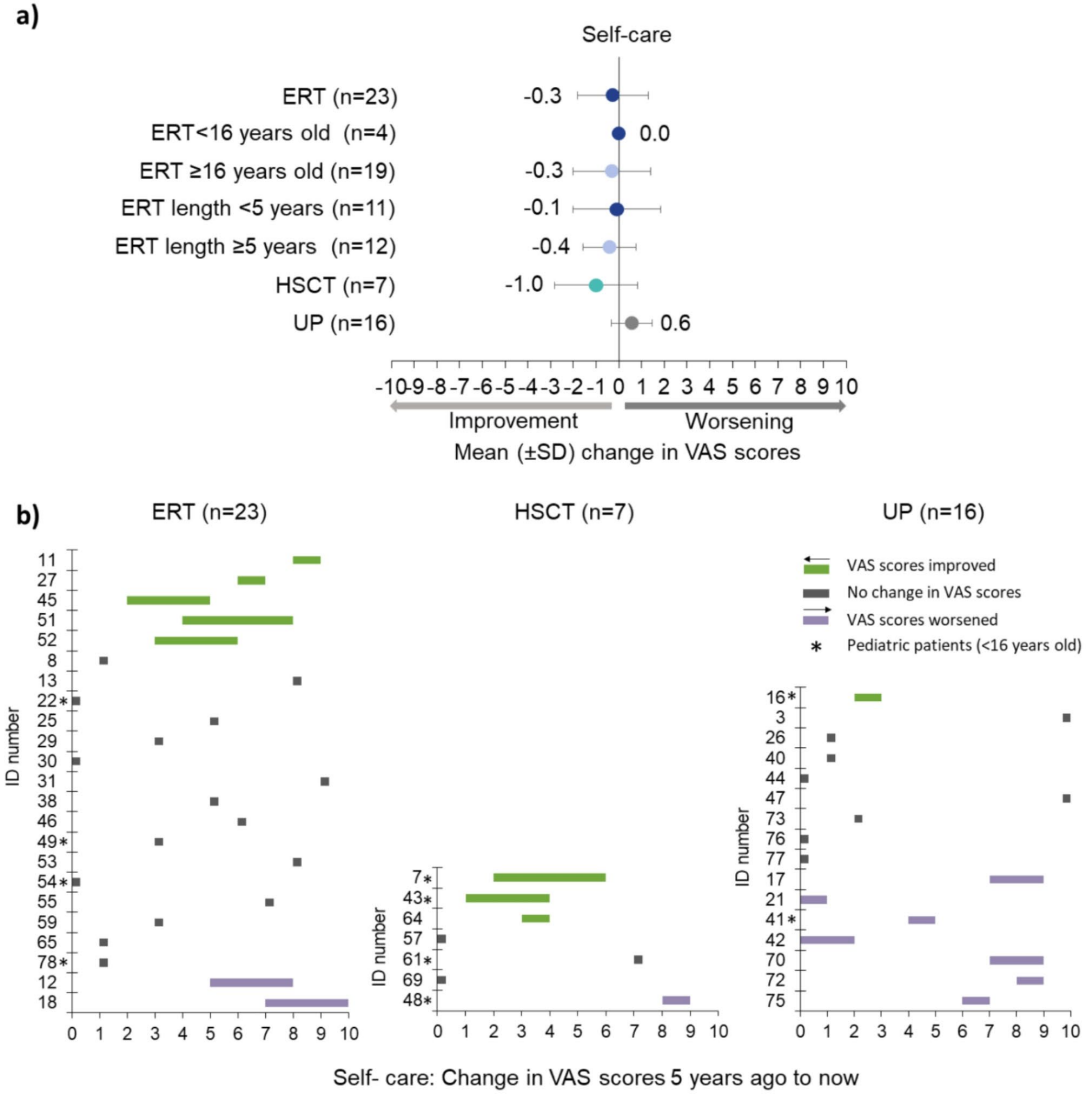
Ability to self-care. Respondents were asked to rate the person with alpha-mannosidosis’ ability to perform self-care activities (e.g., washing, dressing, combing hair, brushing teeth, etc.) now and 5 years ago on a VAS scale, where 0 was being ‘able to do these activities completely independently’ and 10 was being ‘unable to do these activities for themselves, needing complete assistance’. **(a)** mean (± SD) change in VAS scores 5 years ago to now stratified by treatment (ERT, HSCT, UP), pediatric (< 16 years old) vs adults (≥ 16 years old) and length of time receiving ERT (< 5 years; ≥5 years). **(b)** Individual changes in VAS scores for ERT, HSCT, and UP. Green shows improvement, grey no change, and purple worsening/decline. ERT = enzyme replacement therapy; HSCT = hematopoietic stem cell transplantation; SD = standard deviation; UP = untreated patients; VAS = visual analogue scale

Five years ago, the ability to self-care mean VAS scores were the worst for patients treated with ERT (4.7 ± 3.1) and HSCT (4.1 ± 3.2), however, there were trends towards improvements in scores over time for HSCT patients (-1.0 ± 1.8) and those on ERT (-0.3 ± 1.5), while there was a slight worsening in scores for UP (+ 0.6 ± 0.9) (Fig. 4a).

The ability to self-care remained stable in pediatric patients on ERT and slightly improved for adults on ERT (-0.3 ± 1.7). There was a trend towards an improvement regardless of the length of time on ERT (Fig. 4a). Ability to self-care only improved for those starting ERT at pediatric age (-0.8 ± 1.6) but not for those starting as adults (+ 0.1 ± 1.5) (Supplementary Table 1).

#### Individual changes

Ability to self-care in UP improved for one pediatric patient (-1), remained stable for 50.0% (8/16) of patients (all adults) and declined for 43.8% (7/16) (Fig. 4b UP).

*“He was pretty much independent up until the age of roughly 15 […] As his stability deteriorated*,* he needed more and more help with all normal tasks during the day […] He is now entirely dependent on us. I have to get his clothes ready in the morning and help him dress and get his breakfast ready*,* but he is able to eat that himself […].”* Adult UP.

For those treated with HSCT, the ability to self-care improved for three patients, stabilized for another three and worsened for one patient (Fig. 4b HSCT).

The ability to self-care improved for 21.7% (5/23) ERT patients, remained stable for 69.6% (16/23), and worsened for two (Fig. 4b ERT). Out of the 16 patients who had reported no change over time, nine were on ERT for ≤ 6 years and seven for ≥ 10 years (Supplementary Fig. 3a). Two of the five adult patients who improved over time started ERT at pediatric age and three as adults (Supplementary Fig. 3b). The two adults who reported a decline started ERT in their fourth decade of life and had been on ERT for < 5 years (Supplementary Fig. 3a, b). Examples of the experiences of individual patients, reported by patients or caregivers, are highlighted in Supplementary Table 2.

### Mental health

#### Overall changes

**Fig. 5:**
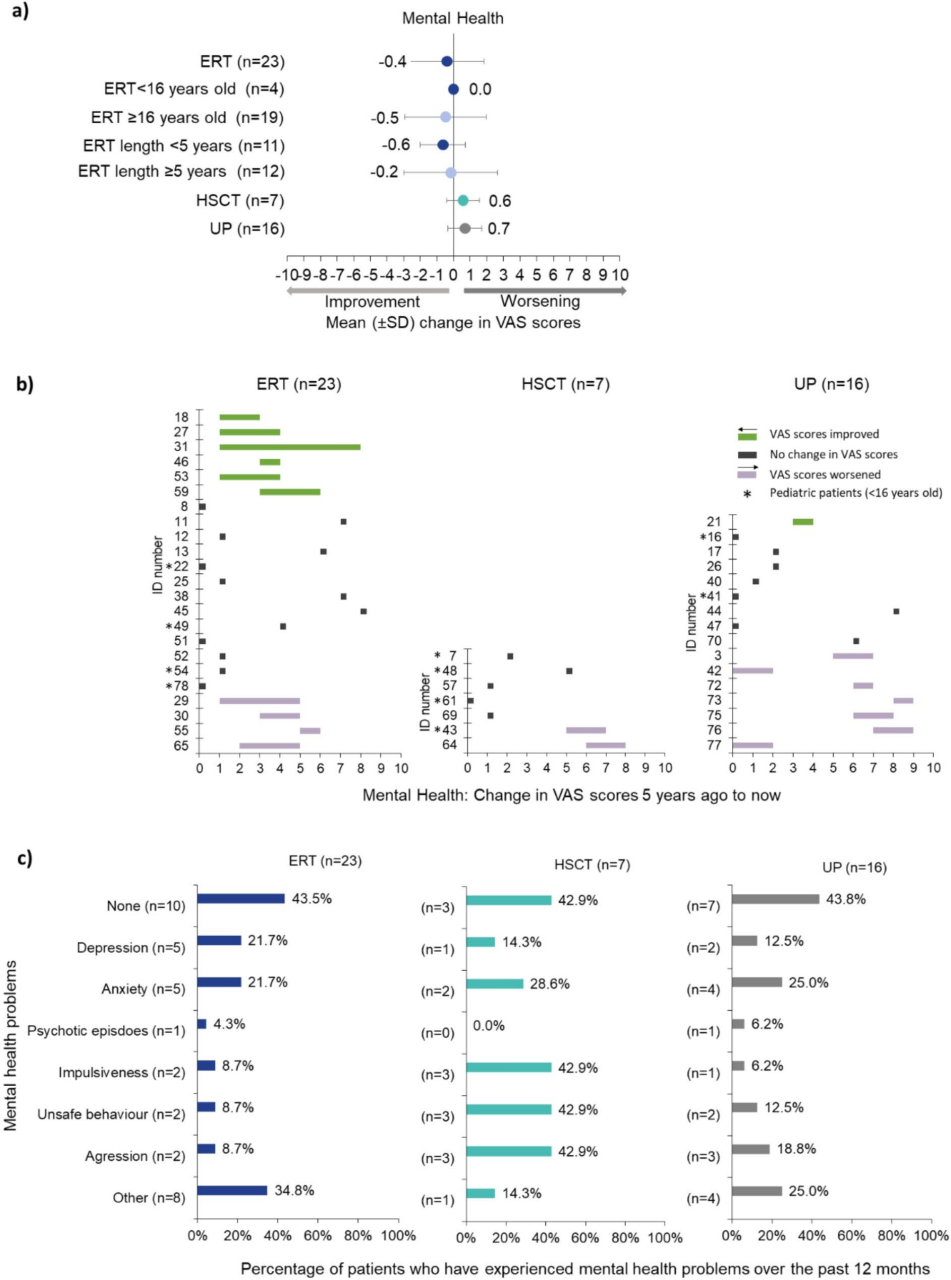
Mental health. Respondents were asked to rate the mental health of the person with alpha-mannosidosis on a VAS scale now and 5 years ago, where 0 was ‘excellent mental health’ and 10 was ‘poor mental health’. **(a)** Mean (± SD) change in VAS scores 5 years ago to now stratified by treatment group (ERT, HSCT, UP), pediatric (< 16 years old) vs adults (≥ 16 years old) and length of time receiving ERT (< 5 years; ≥5 years). **(b)** Individual changes in VAS scores reported by patients showing scores 5 years ago and now for ERT, HSCT, and UP. Green shows improvement, grey no change, and purple worsening/decline; **(c)** Percentage of mental health issues reported by patients by treatment over the past 12 months: ERT (*n* = 23), HSCT (*n* = 7), and UP (*n* = 16). Respondents could select as many responses as applied. Hence, percentages are calculated as number of patients who chose this option over the number of total patients who responded to this question. ERT = enzyme replacement therapy; HSCT = hematopoietic stem cell transplantation; SD = standard deviation; UP = untreated patients; VAS = visual analogue scale

Five years ago, patients treated with HSCT had the best mean mental health VAS score (2.9 ± 2.4), while mean scores for UP (3.4 ± 3.1) and those on ERT (3.3 ± 2.7) were similar (Supplementary Table 1). Over time, only patients receiving ERT slightly improved (-0.4 ± 2.2), resulting in the best mean mental health scores now (2.9 ± 2.7), while UP declined to have the worst mental health scores (4.1 ± 3.4) (Fig. 5a).

Mental health mean scores did not change in pediatric patients on ERT and slightly improved for the adult group (-0.5 ± 2.4). There was a trend towards improvement in scores regardless of whether patients were on ERT for < 5 or ≥ 5 years (-0.6 ± 1.4 and -0.2 ± 2.8, respectively) (Fig. 5a). Mental health worsened for the group of patients who started ERT at pediatric age (+ 0.7 ± 1.1) and largely improved for those who started ERT as adults (-1.1 ± 2.5) (Supplementary Table 1).

#### Individual changes

Among UP, mental health improved for one adult (-1), remained stable for 50.0% (8/16) of patients and declined for 43.8% (7/16) (Fig. 5b UP).

*“My daughter’s quality of life changed initially due to the worsening imbalance but mostly due to the signs and symptoms of depression and anxiety demonstrated with crying and sadness. When asked*,* she denies any symptoms but continues to cry.”* Adult UP.

None of the seven HSCT patients reported an improvement in their mental health but five reported being stable and two reported worsening (Fig. 5b HSCT).

Mental health improved for 26.1% (6/23) of ERT patients, remained stable for 56.5% (13/23) and worsened for 17.4% (4/23) (Fig. 5b ERT). The two adult patients whose mental health scores declined by + 3 and + 4 were on ERT for nearly 6 years (Supplementary Fig. 4a). Adults reporting an improvement in mental health started treatment at 16 years old or above (Supplementary Fig. 4b) but the length of time on treatment varied from around 2 years to over 12 years (Supplementary Fig. 4a). Examples of the experience of individual patients, reported by patients or caregivers, are highlighted in Supplementary Table 2.

#### Type of mental health issues

Respondents were asked what type of mental health issues they had experienced over the past 12 months (Fig. 5c). Overall, just under half of UP (7/16) did not experience any mental health problems, four experienced anxiety, and three aggression. Under ‘other’, patients listed delay in cognitive and language development, lack of confidence, social isolation, and nervousness. Of the seven HSCT patients, three did not experience any mental health problems, however impulsiveness, unsafe behavior, and aggression were reported by three patients each. For those receiving ERT, 43.5% (10/23) of patients did not experience any mental health problems, five experienced depression, and five anxiety. Patients also listed nervousness, feeling upset, cognitive delay, mild impulsivity, anger, child not understanding their own condition, and obsession. Four patients on ERT, two treated with HSCT and three UP received medication for mental health issues.

## Discussion

This survey of 51 individuals with alpha-mannosidosis from 18 countries is the first international study based on patient and caregiver-reported responses covering changes in both functional and QoL domains over 5 years. A comprehensive natural history dataset for alpha-mannosidosis is currently lacking due to the absence of standard medical care and monitoring protocols. Patients can present with a broad range of symptoms and disease severity, which inevitably further complicates data collection and analysis [9]. Although well-defined cut-offs for VAS have not been established, changes in patient health reported by caregivers and patients, such as patient reported outcomes (PROs), are informative and add crucial insight into disease progression not measured in clinical assessments. The results of this study offer vital information not only to inform trends in disease progression in patients with alpha-mannosidosis, but to understand how disease burden affects patient QoL.

Treatment goals focus on slowing or halting the evolution of disease with potential symptomatic or mental health benefits especially when therapy is started early. Given the severity and heterogenous nature of alpha-mannosidosis, such effects can have a meaningful impact on patients QoL. Our results showed variability in individual VAS scores regardless of the treatment status of patients, however, they suggest that early intervention with HSCT or ERT may plausibly delay disease progression, reflecting similar observations in other studies [17–19, 29]. Our study found that patients treated with HSCT or ERT reported less deterioration on all functional and QoL variables investigated compared with UP. Mean scores of HSCT patients improved the most in their ability to self-care with only a slight decline reported in walking ability, compared to ERT and UP patients; for ERT patients, pain and discomfort, ability to self-care, and mental health improved over time. These results concur with Adam et al., showing that from six patients, only those who received HSCT or ERT, as opposed to supportive care, experienced an improvement in health-related QoL (HRQoL) [9]. They are also in accordance with Borgwardt et al., reporting a decrease from baseline in pain and mobility issues, and an improvement undertaking usual activities following ERT [17]. Furthermore, marked differences in VAS scores between those on ERT treatment for less than 5 years versus 5 years or more across all domains were not observed in our study, but showed similar trends to the overall patients on ERT. Data from clinical trials evaluating long-term efficacy and safety of velmanase alfa found that after 12 months, patients already showed improvements in functional measures that were sustained for up to 4 years [17–19, 30].

Our findings suggest benefits of receiving HSCT or starting ERT treatment in childhood. Overall patients starting ERT during childhood reported a slower decline in walking ability than those starting ERT as adults. Similarly, scores for the overall ERT pediatric patients remained the same or slightly improved across all four variables, demonstrating potential slowing of the natural progression of the disease and benefits for future management in adulthood. Our findings concur with ERT clinical trial results showing an improvement in the pediatric subgroup of patients in motor proficiency measured by the Bruininks-Oseretsky (BOT-2) test [19], in the 3-minute stair climb test (3MSCT) [18] and in the disability index (CHAQ DI) [17] if compared to the adult subgroup. Similarly, HSCT patients in our study received the transplantation before 8 years old and showed a lesser decline in all functional and QoL variables compared to UP. This supports post-HSCT studies reporting mannosidase activity within normal ranges and neurodevelopmental progress in HSCT patients which would otherwise rapidly decline in untreated patients over the first decade of life [10, 11, 31]. However, it must be acknowledged that the sample of HSCT patients in our study is smaller and younger overall than those of the untreated and ERT patients, and while results are encouraging, further studies, including a larger sample of patients and longer follow-up times, are required to draw clear conclusions. Despite this, our results suggest a treatment benefit of HSCT if performed in young symptomatic children, concurring with published studies [2, 4].

The age of patients and disease severity in patients receiving different treatments should be considered when interpreting these findings. Children presenting with more severe disease at a young age may have been selected for HSCT, or conversely, rejected for transplantation. Since this study did not collect the clinical history of patients, we cannot determine differences in severity between groups. However, there were differences in the median age of first symptoms and age of diagnosis between the treated and untreated groups of patients. HSCT and ERT patients reported a median age of 0.7 and 1.5, respectively, for age of first symptoms while UP reported a median age of 2.4 years, suggesting symptoms may have been initially less severe in UP. Similarly, diagnosis was confirmed later in the UP group than in the treated groups.

Overall mean walking ability declined regardless of treatment, likely due to disease progression but was greater in UP than in those who received HSCT or were on ERT. The type of walking assistance required over time differed; while treated and UP required more use of walking aids (e.g., crutches, cane, walking frame, help from caregiver), UP now also required wheelchairs or mobile scooters. Single center studies on a small sample of patients (*n* = 9 [32]; *n* = 12 [33]) following the natural course of the disease have shown ataxia and being wheelchair bound as more frequent in the second and third decades of life [2], so a deterioration in walking should not only be more pronounced in UP, but also expected for patients on treatment who reach this age. A decline in walking ability may not be so pronounced in those treated with HSCT because they are below the age at which these problems start to manifest and they received HSCT very early in life not allowing time for these issues to develop. Indeed, the mean walking ability VAS score for the HSCT group was the lowest at baseline (5 years ago).

Equally, age may have played a role in the reported improvement in self-care for HSCT patients as, over 5 years, children may have learnt to manage self-care activities better, something that may also be reflected in the improved scores in pediatric patients on ERT over time versus adults on ERT. There are few long-term studies of patients receiving HSCT, but a study of 15 patients undergoing HSCT reported similar findings, with functional and developmental progress at 2.1 to 12.6 years after transplantation [11]. At a median of 10.4 years after transplant, patients who had received an HSCT in our study still showed a slower disease progression than UP.

Mental health results reported in the survey showed slight improvement only for those receiving ERT, and it is possible that those who had just received treatment and those who were receiving treatment now, might have felt more positive and hopeful than those who were not. For example, HSCT patients had the best mean mental health score of all groups 5 years ago, nearer the time of transplant, while now, the largest improvement in mean scores only occurred in those receiving ERT treatment at the time of the study, with those who had been on ERT for < 5 years showing the largest improvement. Although caregivers of pediatric ERT patients reported that their mental health had remained stable, mental health in adults and in those starting ERT as adults improved, while mental health worsened for patients that began ERT as children. Overall, 43.5% (10/23) of ERT patients had not experienced any mental health problems over the last 12 months. Regular patient management and follow-up programs are usually part of receiving disease specific treatment, something adult patients may feel more positive about than children or UP. Since pain only improved in ERT patients, they may have experienced better mental health due to the reduction in pain. The relationship between pain and mental health has been well documented [34, 35], and reduced pain has been shown to improve mental health, along with other factors contributing to HRQoL [36].

While more research is needed to understand the lack of improvement of mental health in pediatric patients, potential contributing factors may include transition into adulthood, feelings of isolation, and the realization of living with alpha-mannosidosis. As children transition into adulthood, they naturally seek more independence which is significantly limited by both the disease and the burden of receiving treatment. Long-term therapy can cause both emotional and physical exhaustion, making it difficult to cope with the challenges of growing up. Patients diagnosed with a long-term health condition experience higher incidences of mental health problems, including cognitive impairment, anxiety, and depression [37]. Psychiatric symptoms, especially depression and psychotic manifestations may appear in the second or third decades of life in as many as 25% of individuals, and are probably one of the main causes of deterioration in patient and caregiver QoL [32, 38–40].

Although this survey achieved a good overall response rate for a rare disease, data should be interpreted with caution. The small sample size restricted the ability to perform statistically significant subgroup analyses and hindered the ability to make firm conclusions beyond descriptive trends for those groups of small sample size (i.e., HSCT, adult ERT patients). In addition, the sample size for patients on ERT (*N* = 26) was notably larger than that for patients treated with HSCT (*N* = 7) and UP (*N* = 18); individual variation in patient/caregiver-reported data is likely to have a greater impact in smaller samples of patients. VAS scores provide a quantitative measure for each parameter, and although interpretation of results can be challenging without a defined cut-off for a significant change in VAS scores, they are valuable and informative as timepoint measures. It should be noted, however, that in the current study, mean changes in VAS scores were generally small (< 1) and therefore only indicate possible trends towards an improvement or worsening. Recall bias of participants remembering past events 5 years ago could lead to overestimation or underestimation of experiences, but this bias was equally applicable across responses, giving no advantage to any of the treatment groups. It is also worth considering that reporting patient status can be difficult for some variables such as location of pain, especially for individuals with severe intellectual disabilities or young children, who lack the ability to effectively communicate their discomfort, while additional measures may be needed to understand changes in functions involving complex subdomains (e.g., mental health).

## Conclusion

In this first-of-a-kind study, our results provide patient/caregiver-reported insights on the deterioration in walking ability, pain/discomfort, self-care and mental health over 5 years in UP with alpha-mannosidosis and suggest treatment with ERT or HSCT may slow down this natural progression of the disease. Treated patients showed a different disease trajectory over time than patients who solely received supportive care and management of symptoms as they appeared.

While collecting objective clinical data over time was beyond the scope of this project, it remains a promising area for future research initiatives. The results of our survey, based on perceptions of change rather than objective measures, offer a valuable perspective to complement objective clinical data by providing meaningful insights into disease evolution from a patient perspective. This study could inform the natural trajectory of the disease and its characterization to serve as a comparator when designing interventional studies and to better recognize patients and caregivers’ needs and the most appropriate course of care.

## Electronic supplementary material

Below is the link to the electronic supplementary material.

**Additional File 1:** Recruitment and ethical considerations (.docx).

**Additional File 2: Supplementary Table 1**. Mean VAS scores now and 5 years ago and mean change in VAS scores for walking ability, pain, self-care and mental health (.docx).

**Additional File 3: Supplementary Table 2**. Quotes from patients and caregivers on patient experience (.docx).

**Additional File 4: Supplementary Fig. 1**. Change in individual patient’s walking ability VAS scores overtime and (a) length of time on ERT treatment; (b) age at which ERT treatment started (.docx).

**Additional File 5: Supplementary Fig. 2**. Change in individual patient’s pain or discomfort VAS scores overtime and (a) length of time on ERT treatment; (b) age at which ERT treatment started (.docx).

**Additional File 6: Supplementary Fig. 3**. Change in individual patient’s self-care VAS scores overtime and (a) length of time on ERT treatment; (b) age at which ERT treatment started (.docx).

**Additional File 7: Supplementary Fig. 4**. Change in individual patient’s mental health VAS scores overtime and (a) length of time on ERT treatment; (b) age at which ERT treatment started (.docx).

**Additional File 8:** Plain language summary of manuscript.

## Data Availability

The raw dataset is not publicly available to ensure the protection of patient confidentiality within the small population of alpha-mannosidosis patients.
