# Supplementary material for: Evolution of mobility, pain/discomfort, self-care, and mental health in patients with alpha-mannosidosis: an international caregiver and patient survey
Source: Orphanet J Rare Dis. 2025 May 7;20:217. doi: 10.1186/s13023-025-03694-4 (PMC12057280; doi:10.1186/s13023-025-03694-4)
Supplement: Supplementary file 1 — Additional File 1: Recruitment and ethical considerations (.docx). [file 13023_2025_3694_MOESM1_ESM.docx]

**Additional file 1.**

**Recruitment**

Relevant clinicians from specialized centers and LSD/mucopolysaccharidoses (MPS) patient organizations (POs) were identified through the UK MPS Society and Rare Disease Research Partners (RDRP), including authors of major published studies, genetic centers, clinicians’ referrals, and the study sponsor’s contacts.

**Ethical considerations**

No personally identifiable data were collected, survey responses were fully pseudonymized and quotes from participants who did not consent to share direct quotes were paraphrased.
