# Supplementary material for: Evolution of mobility, pain/discomfort, self-care, and mental health in patients with alpha-mannosidosis: an international caregiver and patient survey
Source: Orphanet J Rare Dis. 2025 May 7;20:217. doi: 10.1186/s13023-025-03694-4 (PMC12057280; doi:10.1186/s13023-025-03694-4)
Supplement: Supplementary file 2 — Additional File 2: Supplementary Table 1. Mean VAS scores now and 5 years ago and mean change in VAS scores for walking ability, pain, self-care and mental health (.docx). [file 13023_2025_3694_MOESM2_ESM.docx]

**Additional file 3.**

**Supplementary Table 2.** Quotes from patients and caregivers on patient experience.

| **Walking ability** |
| --- |
| *“Her ability to walk increased as she grew older, however the chances of trips and falls also increased alongside tiredness and pain.”* HSCT patient |
| *“Until 23 months, the child was not able to walk, and he would often fall over. He had the transplant at [around 3.5 years old]. Since then, a significant improvement could be seen. He enjoys walking longer distances but sometimes would still stumble.”* HSCT patient |
| *“Walking has become easier since ERT. Prior to this I needed a wheelchair when my knees and ankles were inflamed after walking too much.”* Adult ERT patient; started treatment as an adult |
| *“She is starting limping […] this was due to the lower proximity bone issues that became more prominent as she grew. At the age of 9 […] started to use a wheelchair for longer distances and uses a step aid if she needs to climb high steps like getting into a high car.”* Pediatric ERT patient; <5 years on treatment |
| **Pain or discomfort** |
| *“[…] she started using a TENS machine which reduces her consumption on painkillers. We often think about the chances of her being in pain when organizing daily activities. This greatly limits us on what activities we can do for the day, who we can encounter and what type of family vacations we are going to have.”* HSCT patient |
| *“He had pain in his teeth, head, hands, back, and legs. After receiving (for almost 2 years) the enzyme substitution, his situation has improved. He is in a better general condition […] he has more strength in his hands and legs.”* Adult ERT patient, started treatment as an adult |
| *“[…] at the age of 13–14 years, when the disease progressed, his knees and feet hurt. He complained about his legs.”* Adult ERT patient, started treatment as a child |
| **Ability to self-care** |
| *“Lived independently for 15 years in a rented apartment in the municipality, the counsellor visits every 2 weeks. Goes to work 3 days a week and to the clubhouse 2 days a week. Has several friends to meet. Meet relatives with dad, friends and travels with mom. Generally social and humorous […].”* Adult UP |
| *“I have learned many things and have become more independent […].”* Adult HSCT patient |
| *“Despite improvements […] she needs help and support getting up and going to bed. Every time you go to the toilet. When dressing and undressing the lower extremities. When showering, preparing meals, shopping (groceries and clothing), visiting doctors and therapists, transport to and from work, as well as planning, designing, and implementing all leisure activities. […] Based on our experience, we would like to state that if enzyme replacement therapy had been available in early childhood, some symptoms would not have occurred at all or only to a lesser extent (hip), which would have led to a higher quality of life.”* Adult ERT patient, started treatment as an adult |
| **Mental health** |
| *“Since approaching adulthood, his mental health issues have gotten worse. He also become increasingly socially disadvantaged. The current situation has become extremely terrible, where the family's main priority has become keeping him stable.”* Adult HSCT patient. |
| *“When he started using aids, he was ashamed of people’s looks and didn’t want to go out, then with the support of the family and the psychologist it seems to have overcome this shame. His activities have remained the same with more effort from those who have to manage it both at home and outside.”* Adult ERT patient; started treatment as an adult |
| *“[…] He couldn’t play football like the other boys or other sports, which isolated him from his friends [...] He has trouble making friends in his [Place name] class because he finds them "odd" […].”* Pediatric ERT patient, on ERT treatment for ≥5 years |
| *“There was improvement with the abdominal pains which were quite frequent 10 years ago. These pains affected his behavior, which was more aggressive.”* Adult ERT patient, started treatment as an adult |
