# Supplementary material for: Evolution of mobility, pain/discomfort, self-care, and mental health in patients with alpha-mannosidosis: an international caregiver and patient survey
Source: Orphanet J Rare Dis. 2025 May 7;20:217. doi: 10.1186/s13023-025-03694-4 (PMC12057280; doi:10.1186/s13023-025-03694-4)
Supplement: Supplementary file 4 — Additional File 4: Supplementary Fig. 1. Change in individual patient’s walking ability VAS scores overtime and (a) length of time on ERT treatment; (b) age at which ERT treatment started (.docx). [file 13023_2025_3694_MOESM4_ESM.docx]

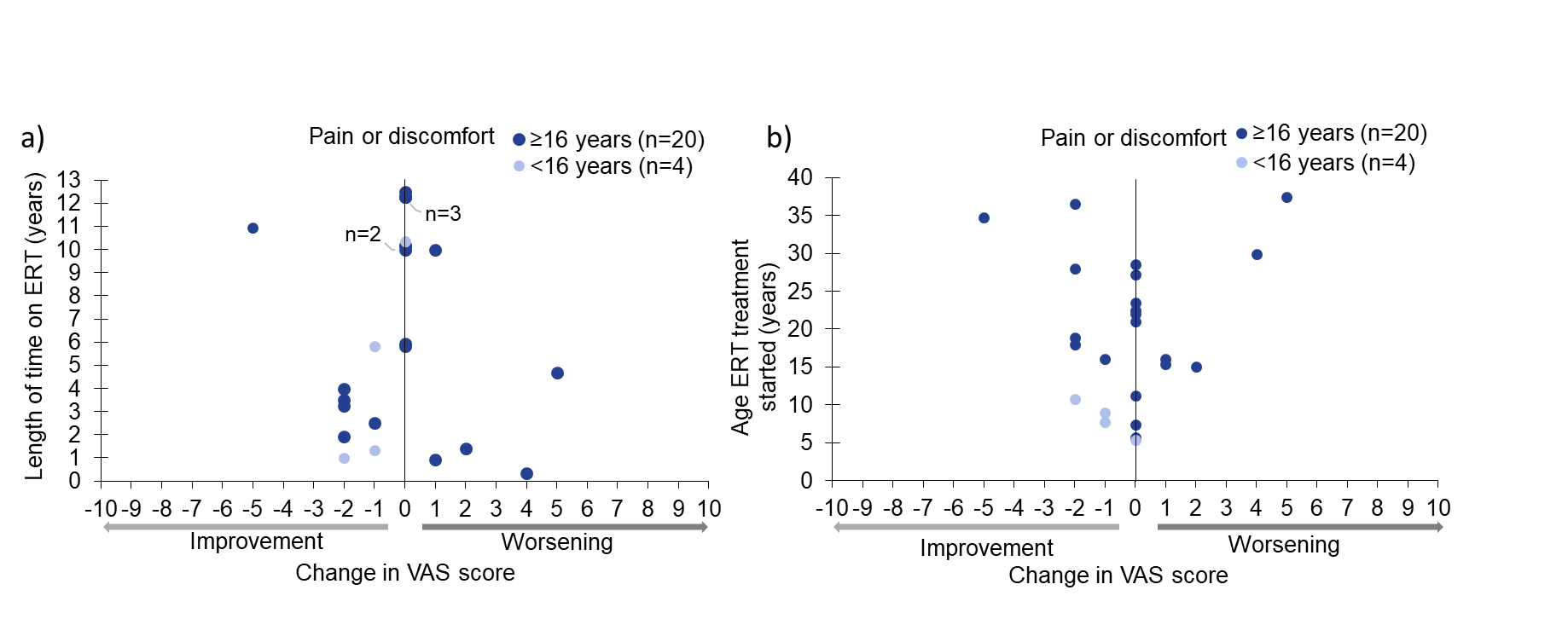
**Additional file 5.**

**Supplementary Figure 2.** Change in individual patient’s pain or discomfort VAS scores overtime and a) length of time on ERT treatment; b) age at which ERT treatment started**.**

*ERT=enzyme replacement therapy; VAS=visual analog scale*
