# Supplementary material for: Evolution of mobility, pain/discomfort, self-care, and mental health in patients with alpha-mannosidosis: an international caregiver and patient survey
Source: Orphanet J Rare Dis. 2025 May 7;20:217. doi: 10.1186/s13023-025-03694-4 (PMC12057280; doi:10.1186/s13023-025-03694-4)
Supplement: Supplementary file 6 — Additional File 6: Supplementary Fig. 3. Change in individual patient’s self-care VAS scores overtime and (a) length of time on ERT treatment; (b) age at which ERT treatment started (.docx). [file 13023_2025_3694_MOESM6_ESM.docx]

**Additional file 7.**


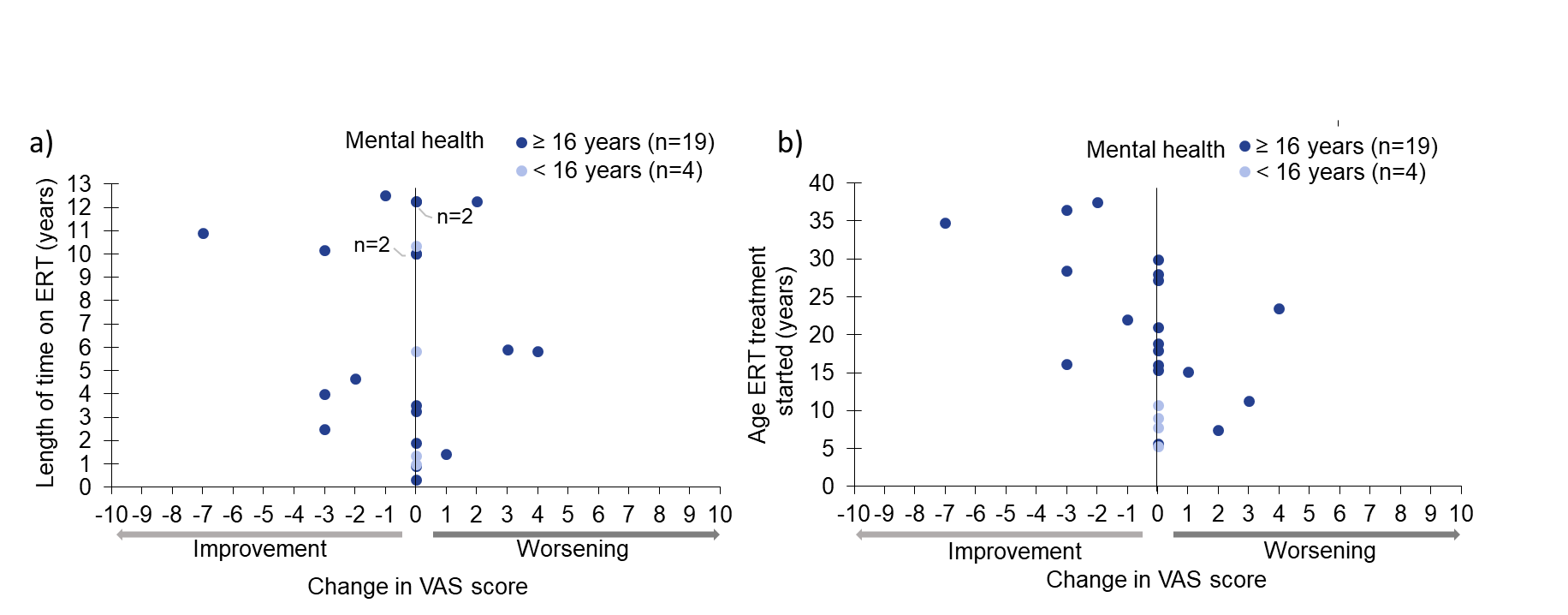


**Supplementary Figure 4.** Change in individual patient’s mental health VAS scores overtime and a) length of time on ERT treatment; b) age at which ERT treatment started.

*ERT=enzyme replacement therapy; VAS=visual analog scale.*
