# Supplementary material for: Evolution of mobility, pain/discomfort, self-care, and mental health in patients with alpha-mannosidosis: an international caregiver and patient survey
Source: Orphanet J Rare Dis. 2025 May 7;20:217. doi: 10.1186/s13023-025-03694-4 (PMC12057280; doi:10.1186/s13023-025-03694-4)
Supplement: Supplementary file 7 — Additional File 7: Supplementary Fig. 4. Change in individual patient’s mental health VAS scores overtime and (a) length of time on ERT treatment; (b) age at which ERT treatment started (.docx). [file 13023_2025_3694_MOESM7_ESM.docx]

**Additional file 2.**

**Supplementary Table 1.** Mean VAS scores now and 5 years ago and mean change in VAS scores for walking ability, pain, self-care and mental health. Length of time on treatment varies for all patients irrespective of treatment group hence data from 5 years ago to now correspond to different follow up periods in relation to start of treatment.

|  | **ERT** | | | | | | | | | | **HSCT** | | **UP** | |
| --- | --- | --- | --- | --- | --- | --- | --- | --- | --- | --- | --- | --- | --- | --- |
|  | All | | Pediatric <16 years old | | Adults  ≥16 years old | <5 years on ERT | ≥5 years on ERT | Started ERT at pediatric age (<16 years old) | Started ERT at adult age  (≥16 years old) | All | | All | |  |
| N | 26 | | 6 | | 20 | 13 | 13 | 11 | 15 | 7 | | 18 | |  |
| **Age at diagnosis** | | | | |  |  |  |  |  |  |  | |  | |
| Median | | 6.6 | | | 7.7 | 6.1 | 7.0 | 6.2 | 7.4 | 4.0 | 2.8 | | 10.3 | |
| Mean±SD | | 8.3±7.2 | | | 7.3±2.1 | 8.6±8.1 | 8.5±7.3 | 8.0±7.3 | 7.5±3.1 | 8.8±9.2 | 3.8±3.0 | | 11.3±7.0 | |
| Range | | 0.0–27.2 | | | 4.5–9.6 | 0.0–27.2 | 1.3–27.2 | 0.0–25.2 | 2.5–14.0 | 0.0–27.2 | 1.3–9.8 | | 2.6–24.1 | |
| **Age treatment started** | | | | |  |  |  |  |  |  | |  | |  |
| Median | 17.0 | | | 8.4 | 21.5 | 16.1 | 21.0 | 9.0 | 23.5 | 3.2 | |  | |  |
| Mean±SD | 18.9±9.7 | | | 9.2±3.3 | 21.8±9.1 | 19.8±10.0 | 17.9±9.7 | 10.0±3.7 | 25.4±7.1 | 4.0±2.4 | |  | |  |
| Range | 5.3–37.5 | | | 5.3–14.8 | 5.7–37.5 | 7.7–37.5 | 5.3–34.8 | 5.3–15.3 | 16.0–37.5 | 1.6–7.8 | |  | |  |
| **VAS scores** |  | | |  |  |  |  |  |  |  | |  | |  |
| **Walking ability** |  | | |  |  |  |  |  |  |  | |  | |  |
| N | 26 | | | 6 | 20 | 13 | 13 | 11 | 15 | 7 | | 18 | |  |
| *5 years-ago* |  |  |  |  |  |  |  |  |  |  |  |  |  |  |
| Median | 4.0 | | | 0.5 | 5.0 | 4.0 | 4.0 | 0.0 | 6.0 | 3.0 | | 4.0 | |  |
| Mean±SD | 3.8±3.2 | | | 1.8±2.6 | 4.4±3.2 | 3.8±3.0 | 3.8±3.5 | 1.3 ±2.0 | 5.7 ±2.6 | 3.1±3.6 | | 3.8±3.2 | |  |
| Range | 0–9 | | | 0–6 | 0–9 | 0–8 | 0–9 | 0–6 | 0–9 | 0–8 | | 0–10 | |  |
| *Now* |  |  |  |  |  |  |  |  |  |  |  |  |  |  |
| Median | 4.5 | | | 1.0 | 6.5 | 4.0 | 6.0 | 1.0 | 8.0 | 3.0 | | 7.0 | |  |
| Mean±SD | 4.5±3.6 | | | 1.8±2.1 | 5.4±3.6 | 4.6±3.5 | 4.5±4.0 | 1.5 ±1.9 | 6.8 ±2.9 | 3.3±3.0 | | 5.6±3.7 | |  |
| Range | 0–10 | | | 0–5 | 0–10 | 0–10 | 0–10 | 0–5 | 0–10 | 0–8 | | 0–10 | |  |
| *Change in VAS scores* | |  |  |  |  |  |  |  |  |  |  |  |  |  |
| Median | 0.5 | | | 0.0 | 1.0 | 1.0 | 0.0 | 0.0 | 1.0 | 0.0 | | 1.5 | |  |
| Mean±SD | 0.7±1.2 | | | 0.0±1.1 | 1.0±1.1 | 0.8±1.4 | 0.6±1.0 | 0.2 ±1.0 | 1.1 ±1.2 | 0.1±1.9 | | 1.8±2.0 | |  |
| Range | (-2)–4 | | | (-2)–1 | 0–4 | (-2)–4 | 0–3 | (-2)–2 | 0–4 | (-2)–4 | | 0–6 | |  |
| **Pain or discomfort** | | | |  |  |  |  |  |  |  | |  | |  |
| N | 24 | | | 4 | 20 | 11 | 13 | 9 | 15 | 7 | | 16 | |  |
| *5 years-ago* |  | | |  |  |  |  |  |  |  | |  | |  |
| Median | 2.0 | | | 1.5 | 3.0 | 4.0 | 1.0 | 2.0 | 4.0 | 1.0 | | 1.0 | |  |
| Mean±SD | 3.3±3.0 | | | 1.8±1.7 | 3.7±3.1 | 3.5±1.6 | 3.2±3.8 | 1.4 ±1.3 | 4.5 ±3.1 | 1.6±1.5 | | 2.1±2.5 | |  |
| Range | 0–10 | | | 0–4 | 0–10 | 2–7 | 0–10 | 0–4 | 0–10 | 0–4 | | 0–8 | |  |
| *Now* |  | | |  |  |  |  |  |  |  | |  | |  |
| Median | 2.0 | | | 0.0 | 2.5 | 3.0 | 1.0 | 0.0 | 4.0 | 2.0 | | 2.0 | |  |
| Mean±SD | 3.1±3.3 | | | 0.8±1.5 | 3.6±3.4 | 3.5±3.2 | 2.8±3.5 | 1.3 ±1.7 | 4.2 ±3.6 | 2.4±1.7 | | 3.1±3.3 | |  |
| Range | 0–10 | | | 0–3 | 0–10 | 0–10 | 0–10 | 0–4 | 0–10 | 0–5 | | 0–9 | |  |
| *Change in VAS scores* | | | |  |  |  |  |  |  |  | |  | |  |
| Median | 0.0 | | | -1.0 | 0.0 | -1.0 | 0.0 | 0.0 | 0.0 | 0.0 | | 0.0 | |  |
| Mean±SD | -0.2±2.0 | | | -1.0±0.8 | -0.1±2.2 | 0.0±2.6 | -0.4±1.4 | -0.1 ±1.2 | –0.3 ±2.4 | 0.9±1.2 | | 1.0±2.7 | |  |
| Range | (-5)–5 | | | (-2)–0 | (-5)–5 | (-2)–5 | (-5)–1 | (-2)–2 | (-5)–5 | 0–3 | | (-5)–8 | |  |
| **Self-care** |  | | |  |  |  |  |  |  |  | |  | |  |
| N | 23 | | | 4 | 19 | 11 | 12 | 9 | 14 | 7 | | 16 | |  |
| *5 years-ago* |  | | |  |  |  |  |  |  |  | |  | |  |
| Median | 5.0 | | | 0.5 | 6.0 | 5.0 | 4.5 | 1.0 | 6.0 | 4.0 | | 2.5 | |  |
| Mean±SD | 4.7±3.1 | | | 1.0±1.4 | 5.4±2.7 | 4.9±1.9 | 4.4±3.9 | 2.8 ±3.2 | 5.9 ±2.4 | 4.1±3.2 | | 3.7±3.8 | |  |
| Range | 0–9 | | | 0–3 | 0–9 | 1–7 | 0–9 | 0–8 | 1–9 | 0–8 | | 0–10 | |  |
| *Now* |  | | |  |  |  |  |  |  |  | |  | |  |
| Median | 4.0 | | | 0.5 | 5.0 | 5.0 | 3.5 | 1.0 | 6.0 | 2.0 | | 2.0 | |  |
| Mean±SD | 4.4±3.2 | | | 1.0±1.4 | 5.1±3.0 | 4.8±2.8 | 4.0±3.6 | 2.0 ±2.3 | 5.9 ±2.7 | 3.1±3.5 | | 4.3±4.0 | |  |
| Range | 0–10 | | | 0–3 | 0–10 | 1–10 | 0–9 | 0–7 | 1–10 | 0–9 | | 0–10 | |  |
| *Change in VAS scores* | | | |  |  |  |  |  |  |  | |  | |  |
| Median | 0.0 | | | 0.0 | 0.0 | 0.0 | 0.0 | 0.0 | 0.0 | 0.0 | | 0.0 | |  |
| Mean±SD | -0.3±1.5 | | | 0.0±0.0 | -0.3±1.7 | -0.1±1.9 | -0.4±1.2 | –0.8 ±1.6 | 0.1 ±1.5 | -1.0±1.8 | | 0.6±0.9 | |  |
| Range | (-4)–3 | | | 0–0 | (-4)–3 | (-3)–3 | (-4)–0 | (–4)–0 | (–3)–3 | (-4)–1 | | (-1)–2 | |  |
| **Mental health** |  | | |  |  |  |  |  |  |  | |  | |  |
| N | 23 | | | 4 | 19 | 11 | 12 | 9 | 14 | 7 | | 16 | |  |
| *5 years-ago* |  | | |  |  |  |  |  |  |  | |  | |  |
| Median | 3.0 | | | 0.5 | 4.0 | 4.0 | 2.5 | 2.0 | 4.0 | 2.0 | | 3.0 | |  |
| Mean±SD | 3.3±2.7 | | | 1.3±1.9 | 3.7±2.7 | 3.6±2.7 | 3.0±2.8 | 2.6 ±2.7 | 3.8 ±2.7 | 2.9±2.4 | | 3.4±3.1 | |  |
| Range | 0–8 | | | 0–4 | 0–8 | 0–8 | 0–8 | 0–8 | 0–8 | 0–6 | | 0–8 | |  |
| *Now* |  | | |  |  |  |  |  |  |  | |  | |  |
| Median | 1.0 | | | 0.5 | 3.0 | 1.0 | 2.0 | 4.0 | 1.0 | 2.0 | | 2.5 | |  |
| Mean±SD | 2.9±2.7 | | | 1.3±1.9 | 3.3±2.7 | 3.0±2.8 | 2.8±2.6 | 3.2 ±3.0 | 2.7 ±2.5 | 3.4±3.2 | | 4.1±3.4 | |  |
| Range | 0–8 | | | 0–4 | 0–8 | 0–8 | 0–7 | 0–8 | 0–7 | 0–8 | | 0–9 | |  |
| *Change in VAS scores* | | | |  |  |  |  |  |  |  | |  | |  |
| Median | 0.0 | | | 0.0 | 0.0 | 0.0 | 0.0 | 0.0 | 0.0 | 0.0 | | 0.0 | |  |
| Mean±SD | -0.4 ±2.2 | | | 0.0±0.0 | -0.5±2.4 | -0.6±1.4 | -0.2±2.8 | 0.7 ±1.1 | –1.1 ±2.5 | 0.6±1.0 | | 0.7±1.0 | |  |
| Range | (-7)-4 | | | 0–0 | (-7)-4 | (-3)-1 | (-7)-4 | 0–3 | (–7)–4 | 0-2 | | (-1)-2 | |  |

*ERT=enzyme replacement therapy; HSCT=hematopoietic stem cell transplant; SD=standard deviation; UP=untreated patients; VAS=visual analog scale*
