# Supplementary material for: Evolution of mobility, pain/discomfort, self-care, and mental health in patients with alpha-mannosidosis: an international caregiver and patient survey
Source: Orphanet J Rare Dis. 2025 May 7;20:217. doi: 10.1186/s13023-025-03694-4 (PMC12057280; doi:10.1186/s13023-025-03694-4)
Supplement: Supplementary file 8 — Additional File 8: Plain language summary of manuscript. [file 13023_2025_3694_MOESM8_ESM.pdf]

Evolution of mobility, pain/discomfort, self-care, and mental health in patients with alpha-mannosidosis: an international caregiver and patient survey

Supplemental information to Stepien et al. *Orphanet Journal of Rare Diseases*. 2025

This is a plain language summary of an article about the evolution of the burden of illness and quality of life (QoL) experienced by patients with alpha-mannosidosis, which was published in *Orphanet Journal of Rare Diseases* in 2025.

What is alpha-mannosidosis?

Alpha-mannosidosis (AM) is an extremely rare, inherited condition in which the enzyme called alpha-mannosidase does not work properly or is missing. This enzyme is responsible for helping clear waste from the cells in our body. Without this enzyme, oligosaccharides (complex nutrients that provide the body with energy) build up in the cells, which can make it harder for them to function properly. People with AM can experience a variety of symptoms that affect different organs, and these can become more severe over time.

Why was the study done?

As an ultra-rare disease, there's not much known about how alpha-mannosidosis affects people over time. To learn more, an international survey was conducted with patients and their caregivers. The goal was to better understand how the disease impacts their lives and quality of life (QoL) by comparing their experiences from five years ago to now.

What type of questions were included in the survey?

The people who took part in the survey answered both multiple-choice and open-ended questions. They also rated different aspects of life for someone with alpha-mannosidosis using a **Visual Analog Scale (VAS).** This included ratings on how well they could walk, how much pain or discomfort they felt, their ability to take care of themselves, and their mental health. Comparing the scores from now and five years ago helps show which areas have gotten worse, stayed the same, or improved over the years.

What is a VAS? VAS is a measurement tool where participants mark the intensity of a feeling from none to maximum.

No problem walking about — Unable to walk about

0 1 2 3 4 5 6 7 8 9 10

Now

5 years ago

Example of a Visual Analog Scale (VAS)

What treatments are available for patients with alpha-mannosidosis?

There are few treatments for AM, with some patients only receiving supportive care and management of symptoms as they appear. The two main options are:

1. **Hematopoietic stem cell transplantation (HSCT)** Replaces diseased bone marrow with healthy cells that produce the missing enzyme.
2. **Enzyme replacement therapy (ERT)** Replaces or supplements the missing or impaired enzyme.

Who took part?

51 people from **18** countries completed the survey

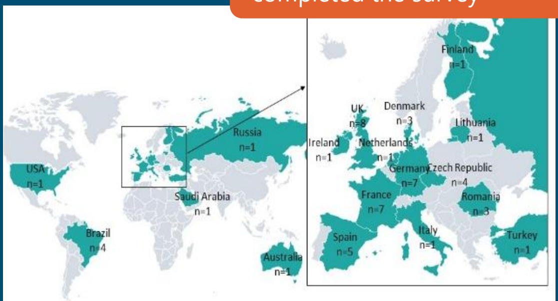

Respondents were mostly parents. Three adults with alpha-mannosidosis also took the survey.

About the survey

Translated into **13 languages**

Completed online between November 2022 & February 2023

The survey asked patients and caregivers about:

- the **impact of living with alpha-mannosidosis**
- how their **overall health and daily lives** had changed **now compared with 5 years ago**

About the individuals with alpha-mannosidosis in this study

Information was collected for **51 children and adults with alpha-mannosidosis**

26 male

25 female

12 children (10–16 years old)

39 adults (16–46 years old)

26 people Receiving ERT

6 children **20** adults

Average age at first symptoms: **3** years → Average age at diagnosis: **8** years → Average age ERT started: **19** years → **Average age now 25 years old**

7 people Received HSCT

4 children **3** adults

Average age at first symptoms: **2** years → Average age at diagnosis: **4** years → Average age they received HSCT: **4** years → **Average age now 16 years old**

18 people Not treated

2 children **16** adults

Average age at first symptoms: **6** years → Average age at diagnosis: **11** years → **Average age now 26 years old**

What do these results mean for patients?

Alpha-mannosidosis is a disease that gets worse over time, a key aim is to slow down and stabilize its progression and to stabilize or improve the patients' everyday lives.

The table below illustrates the average VAS scores of all patients who reported an improvement, a decline, or no change in their health and daily lives.

Patients treated with HSCT or ERT reported less decline on all the areas investigated compared with those who did not get any treatment. However, patients were very variable in their responses, no matter if they had received treatment or not. This shows the variability of the symptoms and the disease.

Since pain only improved in ERT patients, the improvement that can be seen in their mental health may have been due to this reduction in pain.

Potential contributing factors to the variability in mental health responses may include transition into adulthood, feelings of isolation, and the realization of living with alpha-mannosidosis. Long-term therapy can cause both emotional and physical exhaustion, making it difficult to cope with the challenges of growing up.

	Walking ability	Pain/ Discomfort	Self care	Mental health
All patients on ERT (23 patients)	x	✓	✓	✓
Patients on ERT younger than 16 years old (19 patients)	=	✓	=	=
Patients on ERT 16 years old or older (4 patients)	xx	✓✓	✓	✓
Patients who have been on ERT for less than 5 years (11 patients)	x	=	✓	✓
Patients who have been on ERT 5 years or longer (12 patients)	x	✓	✓	✓
Patients who have received HSCT (7 patients)	x	x	✓✓	x
Not treated patients (16 patients)	xx	xx	x	x

The pediatric patients receiving ERT remained the same or slightly improved in their abilities in all the areas that were explored, this could be due to potential slowing of the natural progression of the disease.

HSCT patients showed the most improvement in their ability to self-care and the least decline in walking ability, compared to patients receiving ERT or untreated patients.

The average walking ability declined for all regardless of treatment, but it was greater in those not treated than in those who received HSCT or receiving ERT. For those receiving ERT, pain and discomfort, ability to self-care, and mental health improved over time.

Age may have played a role in the reported improvement in self-care for HSCT patients, children over 5 years may have learnt to manage self-care better. This could also be a reason for the slight improvement in pediatric patients receiving ERT over time.

xx Worse | x Slightly worse | = No change | ✓ Slight improvement | ✓✓ Improvement

*Classifications based on the differences in VAS scores now compared to 5 years before, scores under 1 are classified as slight change

What did this study tell us and why is it important?

1. The results offer valuable insights into how alpha-mannosidosis progresses from the patients' perspective and its impact on their quality of life.

2. Patients who received treatment with ERT or HSCT had a different progression of the disease over time compared to those who only received supportive care and management of symptoms as they appeared.

3. The findings suggest that starting treatment early may help slow down the progression of the disease.

4. This study helps us understand the natural course of the disease and can guide future research. It also highlights the needs of patients and caregivers, ensuring better care options.

Points to consider: Although this study achieved a good overall response rate for a rare disease, the data should be interpreted with caution. The small sample size restricted the analyses and impacted the ability to make firm conclusions beyond descriptive trends.

THANK YOU!

The researchers would like to thank patients and caregivers for their time to participate in the survey and for sharing their experience with us to support this project to understand the progression of alpha-mannosidosis.

This summary was produced by Rare Disease Research Partners on behalf of Chiesi Farmaceutici S.p.A.
